# Fecal Glucocorticoid Metabolites as a Noninvasive Indicator of Stress in the Tsushima Leopard Cats (*Prionailurus bengalensis euptilurus*): Application to Health Care

**DOI:** 10.3390/ani12091072

**Published:** 2022-04-21

**Authors:** Satoshi Kusuda, Takashi Funahashi, Itsuki Adachi, Hanae Yamamoto, Eiji Nagao, Kirito Matsui, Yuki Akiba

**Affiliations:** 1 Laboratory of Animal Reproduction, Faculty of Applied Biological Sciences, Gifu University, Gifu 501-1193, Japan; takashi.funahashi.3412@gmail.com (T.F.); i-adachi@morikirara.jp (I.A.); 2 Saikai National Park Kujukushima Zoo and Botanical Garden, Sasebo 857-1231, Japan; 3 Tsushima Wildlife Conservation Center, Ministry of the Environment, Tsushima 817-1603, Japan; rana.rana.tsushimensis@gmail.com; 4 Fukuoka Zoo and Botanical Garden, Fukuoka 810-0037, Japan; eiji.nagao.30@gmail.com; 5 Yokohama Zoological Gardens ZOORASIA, Yokohama 241-0001, Japan; matsui@hama-midorinokyokai.or.jp; 6 Toyama Municipal Family Park Zoo, Toyama 930-0151, Japan; y-akiba2020@toyama-familypark.jp

**Keywords:** carnivore, cortisol, fecal hormone, glucocorticoid, leopard cat, stress

## Abstract

**Simple Summary:**

The Tsushima leopard cat (*Prionailurus bengalensis euptilurus*) and the Iriomote cat (*P. b. iriomotensis*) are the only two wild felids living in Japan. Both species are endangered. The Tsushima leopard cat is undergoing captive breeding (ex situ conservation) in zoos with the aim of returning it to the wild, in addition to conservation activities in the habitat (in situ conservation). In order to promote captive breeding, it is important to elucidate the reproductive physiology and to evaluate stress conditions. We investigated the possibility of measuring glucocorticoid metabolites (GCMs), which are stress hormones, using feces in Tsushima leopard cats. We examined changes in fecal GCM in individuals who underwent a health examination under anesthesia as an unavoidable stressful condition during normal health care. Fecal GCM concentrations increased 1–2 days after the health examination. Fecal GCM concentrations were higher in diseased individuals than in apparently healthy individuals. Individuals diagnosed with disk herniation also showed a decrease in fecal GCM concentrations after treatment with therapeutics. These results indicate that the measurement of fecal GCMs is useful for improving the husbandry and health management of the Tsushima leopard cat.

**Abstract:**

This study investigates whether the measurement of glucocorticoid metabolites (GCMs) in feces is a useful method for the noninvasive evaluation of stress in the endangered Tsushima leopard cats (*Prionailurus bengalensis euptilurus*). Feces were collected from six seemingly healthy and five diseased (renal dysfunction, adrenal tumor, hernia, feline immunodeficiency virus (FIV), feline leukemia virus (FeLV)) Tsushima leopard cats in captivity. Fecal GCMs were measured by enzyme immunoassay (EIA) for cortisol. Individuals that experienced a physical examination under anesthesia showed increased fecal GCMs 1–2 days after the event. An individual diagnosed with disk herniation showed decreased fecal GCMs after medical administration. The mean fecal GCM concentrations for six healthy animals and five diseased animals were 0.66 ± 0.08 and 2.65 ± 0.76 μg/g, respectively, which was significantly different. Cortisol and corticosterone were not clearly detected in the feces examined by the use of the HPLC-EIA analysis. GCMs may be excreted in the feces; however, the exact identification of these substances is not achieved. The results suggest that the measurement of fecal GCMs is useful for the husbandry and health management of this species.

## 1. Introduction

The Tsushima leopard cat (*Prionailurus bengalensis euptilurus*) is a wild felid inhabiting only Tsushima island, Nagasaki, Japan. The Tsushima leopard cat is considered a regional population of the Amur leopard cat, which is a subspecies of the leopard cat (*P. bengalensis*) [1]. Wild felids can be influenced by environmental changes or decreased prey populations due to the natural dependencies of apex predators in many ecological systems [2]. Therefore, geographical isolation and decreased populations are common trends. The Tsushima leopard cat population was distributed throughout the entire region of Tsushima Island before the 1960s, with an estimated 250–300 individuals [3]. However, a population census by the Japanese Ministry of the Environment in the late 2010s estimated that they decreased to fewer than 90 individuals [4]. The causes of the population decline were roadkill, deforestation, and competition with feral domestic cats. Furthermore, they were not only facing a population decline, but their spatial distribution was shrinking due to their concentration in the northern area of Tsushima Island [3].

The Tsushima leopard cat was designated as a Natural Monument of Japan in the Law for the Protection of Cultural Properties in 1971, and as a National Endangered Species in the Act on Conservation of Endangered Species of Wild Fauna and Flora in 1994. Furthermore, the Tsushima leopard cat was classified as the Critically Endangered species in the Red List of Threatened Species of the Japanese Ministry of the Environment. Accordingly, an enforcement policy was established in 1995, called the “Programmes for Rehabilitation of Natural Habitats and Maintenance of Viable Populations in Tsushima leopard cat”, with aims to maintain stable populations of this species in the wild, and a more concrete plan was settled on in 2010. Recently, the attempts to move individuals between zoos, form new pairs, and assist breeding by artificial insemination [5] have been developed to improve captive breeding and ex situ conservation. However, the movement of individuals and formation of new pairs may cause stress for this species.

Stress involves nonspecific reactions of organisms to ambient stimuli, and a common stress reaction typically exists for various stress stimuli (e.g., restraint, transportation, blood collection, and hypoglycemia) [6]. Various changes to the endocrine response occur in organisms to maintain homeostasis as a response to these stress stimuli [7]; therefore, chronic stress influences growth and sexual maturation [8]. One of the responses to stress stimuli is increased adrenal gland activity through the activation of the hypothalamic–pituitary–adrenocortical axis (HPA axis). When the HPA axis is activated by stress stimuli, the adrenocorticotropic hormone (ACTH) is secreted from the anterior pituitary gland, and the synthesis and secretion of glucocorticoid (GC) from the adrenal cortex is stimulated [9]. GC has anti-inflammatory properties, so the secretion of chronic GC influences the reaction of immune cells and impairs the immunity function in organisms [10]. Additionally, social stress stimuli can be useful in affecting the reproductive physiological state of organisms; however, an inappropriate breeding environment may exert a bad influence on reproduction by decreasing the secretion of the sex hormone and the sensitivity of the ovaries and testes [11,12]. In other words, it is very important to not only investigate endocrine conditions, but also to consider these stress stimuli to promote the reproductive success of rare animals.

Thus far, adrenal activity has been monitored by measuring serum GC concentrations as one of the stress indicators [13], but blood collection itself can elicit a stress response [14]. Serum concentrations of many different hormones also have circadian rhythms, so it should be clarified whether the change in the serum GC concentration is caused by circadian variation or stress stimuli [15,16]. Thus, a method for measuring the amount of fecal GCM, involving an easy sample collection, noninvasive procedures, regular collection intervals, and one that does not have to consider circadian variation, would be useful for monitoring the adrenal activity for stress evaluation. The utility of monitoring adrenal activity by measuring fecal GCMs has been validated in many species [17], and the results of experiments using 14 C-cortisol administered in the veins of domestic cats revealed that 81.9 ± 3.8% of cortisol metabolites was excreted in the feces around 22 ± 6 h later [18,19]. It was reported that the amount of fecal GCMs increased in 1–2 days after the prescription of ACTH in the domestic cat, the cheetah (*Acinonyx jubatus*), the clouded leopard (*Neofelis nebulosi*), the Asian black bear (*Ursus thibetanus*), the sloth bear (*Melursus ursinus*), the black-footed ferret (*Mustela nigripes*), the meerkat (*Suricata suricatta*), and the red wolf (*Canis rufus*) [20]. In the Siberian tiger (*Panthera tigris altaica*), the amount of fecal corticosterone was increased 2 days after being moved between their breeding institutions [21], and the clouded leopard showed that fecal GCMs were affected by the rearing environment [22]. Additionally, surgery caused the amount of fecal GCMs to increase in cheetahs and clouded leopards after their operations [20]. 

Many reports have suggested that the measurement of fecal GCMs is useful as a stress indicator in various carnivores. In small felids, the relationships between the fecal GCM concentration and enclosure size and enrichment have been investigated in the tigrina (*Leopardus tigrinus*) and margay (*L. wiedii*) [23]. However, the fecal GCMs and stress status of the Tsushima leopard cats have not been reported on yet. Therefore, in this study, the variance of fecal GCM concentrations around periods of stress, such as physical examinations and pain from disease, is examined to consider whether there is a difference in fecal GCM concentrations between healthy and diseased individuals and whether these measurements in Tsushima leopard cats are useful for evaluating stress.

## 2. Materials and Methods

### 2.1. Animals and Sample Collection

Six seemingly healthy and five diseased (renal dysfunction, adrenal tumor, hernia, feline immunodeficiency virus (FIV), feline leukemia virus (FeLV)) Tsushima leopard cats were used in this study (Table 1). These animals were kept at Tsushima Wildlife Conservation Center (TWCC), Ministry of the Environment, and 3 zoological institutes, including Fukuoka Zoo and Botanical Garden (FZG), Yokohama Zoological Gardens ZOORASIA (YZG), and Toyama Municipal Family Park Zoo (TFPZ) in Japan. TWCC is a treatment institution for sick and injured individuals and a temporary shelter of wild individuals. 

The Tsushima leopard cats were fed multiple foods, including mice, chick, adult chicken head, broiler chicken, kangaroo meat, beef liver, horse meat, and fish (Japanese horse mackerel), and provided water ad libitum.

All cats were reared individually so that feces were certain to be from only one cat. Fecal samples were collected 1–7 days per week. However, there were rare periods when collection was not possible. Fresh-looking feces were collected from the floor of the enclosure or the ground throughout the study and stored at −20 °C until hormone analysis. Fecal corticosteroids were analyzed by using feces collected before and after the physical examination under anesthesia (physical measurement and blood collection). 

The Tsushima leopard cats were reared in the Japanese Association of Zoos and Aquariums (JAZA) member zoos under an agreement with the Ministry of the Environment. The animals were kept and managed in accordance with JAZA animal welfare regulations. In this study, sample collection (feces) was performed noninvasively during routine husbandry and medical treatment. The fecal samples of the Tsushima leopard cats were obtained with permission from the Kyushu Regional Environment Office of the Ministry of the Environment.

### 2.2. Fecal Glucocorticoid Analysis

Fecal GCMs were extracted using a methanol extraction method [24]. Briefly, frozen feces were dried for approximately 6 h at 100 °C in a forced convection oven (FC-410: Advantec, Tokyo, Japan) and crushed using a hammer. The digested fecal powder was manually separated from indigested hair, bones and grasses. A portion of the fecal powder, 0.1 g, was then extracted with 5 mL of 80% methanol by vortex-mixing for 30 min and left to stand overnight at 4 °C. After centrifugation at 2500 rpm for 10 min, the supernatant (methanol fraction) was transferred to a clean tube and then diluted at a ratio of 1:10 with assay buffer (phosphate buffer containing 0.1% bovine serum albumin) for cortisol assay.

Concentrations of fecal GC metabolites were determined by enzyme immunoassay (EIA) using cortisol (11β,17,21-trihydroxypregn-4-ene-3,20-dione) standard (086-02484, Wako Pure Chemical Industries, Osaka, Japan), cortisol-3-CMO-HRP (dilution of 1:200,000; FKA403, Cosmo Bio, Tokyo, Japan), and anti-cortisol-3-CMO-BSA (dilution of 1:100,000; FKA404-E, Cosmo Bio). The cross-reactivity of the cortisol antibody was 100% for cortisol, 11.5% for 11-deoxycortisol, 4.0% for cortisone, 2.0% for corticosterone, 0.2% for 17α-hydroxy-11-deoxy-corticosterone, and 0.04% for 17α-hydroxy-progesterone from the product data sheet. Serial dilutions of fecal extracts from samples demonstrated parallelism with the cortisol standard curves. Intra- and inter-assay coefficients of variation for cortisol EIA were 4.1% and 10.1%, respectively.

### 2.3. High-Performance Liquid Chromatography (HPLC)

To identify fecal GC metabolites, HPLC was performed using feces of the high concentrations of fecal GC in the Tsushima leopard cat No.52 and No.54. The HPLC sample pretreatment and HPLC protocol were adopted from Adachi et al. [24,25]. Before HPLC, feces were dried and crushed, and 2.0 g of the fecal powder was then extracted with 10 mL of 80% methanol by vortex-mixing for 30 min. After the extract was centrifuged at 2500 rpm under 4 °C for 10 min, the 5 mL supernatant was added to 35 mL assay buffer and the total volume was passed through a Sep-Pak C18 column (Sep-Pak Plus C18, Environmental cartridges, WAT023635, Waters, Milford, MA, USA). GC metabolites were eluted with 5 mL absolute methanol. To separate fecal GC metabolites, a 100 μL extract sample was injected onto the HPLC with reverse-phase Nova-Pak C18 column (3.9 mm × 300 mm, JJAN11695, Waters). For the HPLC, an isocratic solvent of acetonitrile (ACN)/water (H_2_O) (40:60, *v*/*v*) was used at a flow rate of 0.3 mL/min, and 80 fractions (300 μL each) were collected. Each fraction was extracted twice with diethyl ether and inspected by EIAs for cortisol and corticosterone. Cortisol EIA was performed by the protocol mentioned in the preceding paragraph. Corticosterone EIA was performed by using corticosterone (11β,21-dihydroxypregn-4-ene-3,20-dione) standard (037-17583, Wako Pure Chemical Industries, Osaka, Japan), corticosterone-3-CMO-HRP (dilution of 1:200,000; FKA419, Cosmo Bio, Tokyo, Japan), and anti-corticosterone-3-CMO-BSA (dilution of 1:500,000; FKA420-E, Cosmo Bio). The cross-reactivity of the corticosterone antibody was 100% for corticosterone, 8.0% for deoxycorticosterone, 2.1% for progesterone, 0.23% for 11-dehydrocorticosterone, 0.2% for cortisol, 0.05% for 4-androstenedione, 0.05% for cortisone, 0.05% for 17α-hydroxy-11-deoxy-corticosterone, and 0.04% for 17α-hydroxy-progesterone from the product data sheet. Serial dilutions of extracts from fecal samples demonstrated parallelism with the corticosterone standard curve. The immunoreactive fraction numbers were compared with reference tracer elution time, and fecal GC metabolites were identified. The cortisol and corticosterone standards were used as reference tracers, and the elution time of each reference tracer from these reference tracer mixtures was determined by EIA for cortisol and corticosterone. Intra- and inter-assay coefficients of variation for corticosterone EIA were 5.8% and 8.0%, respectively.

### 2.4. Data Analyses

All fecal data were expressed as per gram of dry feces. Data on fecal GC concentrations were presented as the mean ± SEM. The mean of fecal GC concentrations for comparison in healthy and diseased animals was calculated from all data in each animal. To avoid the effects of temporary stress, data from the day of anesthesia to the 4th day were excluded from each mean for the 4 examined animals. Averaged GC concentration differences were compared between healthy and diseased animals with the *t*-test.

## 3. Results

### 3.1. Fecal Glucocorticoid Associated with Physical Examination and Disease

**Figure 1:**
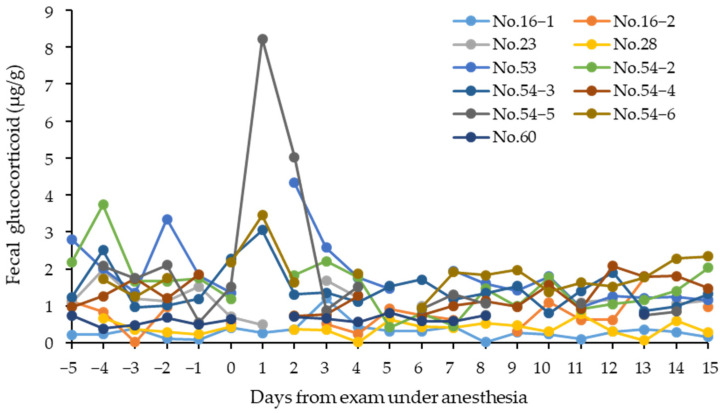
Changes in fecal glucocorticoid metabolite concentrations in 6 Tsushima leopard cats (No.16, No.23, No.28, No.53, No.54, and No.60) measured around the day of physical examination. All cats had their health condition examined under anesthesia. No.52 and No.54−1 were excluded from the figure because feces could not be collected for 4 and 6 days after treatment, respectively.

Increased fecal GCM concentrations were observed in 4 (No.53, No.54−3, No.54−5, and No.54−6) of 11 cases from 6 animals that received physical examination under anesthesia (Figure 1). No.52 and No.54−1 were excluded because feces could not be collected for 4 and 6 days after treatment, respectively. Individuals No.53 and No.54 showed an increased fecal GCM concentration of 4.33 μg/g on the second day (No.53), 3.05 μg/g on the first day (No.54−3), 8.22 μg/g on the first day (No.54−5), and 3.45 μg/g on the first day (No.54−6) after the medical examination under anesthesia, and the amount decreased after that. Feces could not be collected on the following day after treatment in No.16−2, No.28, No.53, No.54−2, and No.60, and on the second day after treatment in No.16−2 and No.23. 

**Figure 2:**
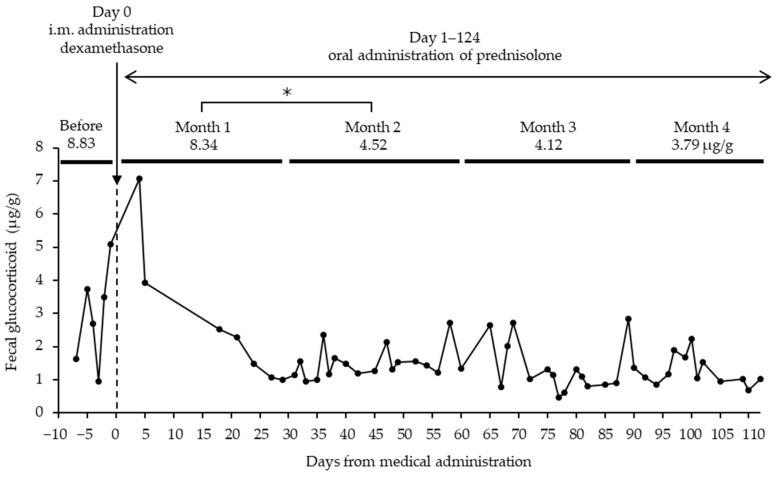
Changes in fecal glucocorticoid metabolite concentrations since the day of dexamethasone administration in a Tsushima leopard cat No.52 diagnosed with disc herniation. For medical treatment, intramuscular injection of dexamethasone and oral administration of prednisolone were administered as anti-inflammatory agents. The prednisolone was intermittently administered during the study period. * Significant difference (*p* < 0.05).

Individual No.52, diagnosed with disc herniation, showed increased fecal GCM concentrations after the symptoms began, and the mean of the amount of fecal GCMs from when the symptoms began through just before treatment was 8.83 ± 1.86 μg/g (Figure 2). Subsequently, dexamethasone, a corticosteroid with anti-inflammatory properties, was administered intramuscularly, and prednisolone, an anti-inflammatory agent, was intermittently administered orally after the beginning of treatment and throughout the GCM measurement period.

The fecal GC concentrations gradually decreased after drug administration. The mean GC levels were 8.34 ± 2.46 μg/g for month one, 4.52 ± 0.33 μg/g for month two, 4.12 ± 0.58 μg/g for month three, and 3.79 ± 0.41 μg/g for month four, significantly lower than month two (*p* < 0.05).

### 3.2. Comparison of Healthy and Diseased Animals

**Figure 3:**
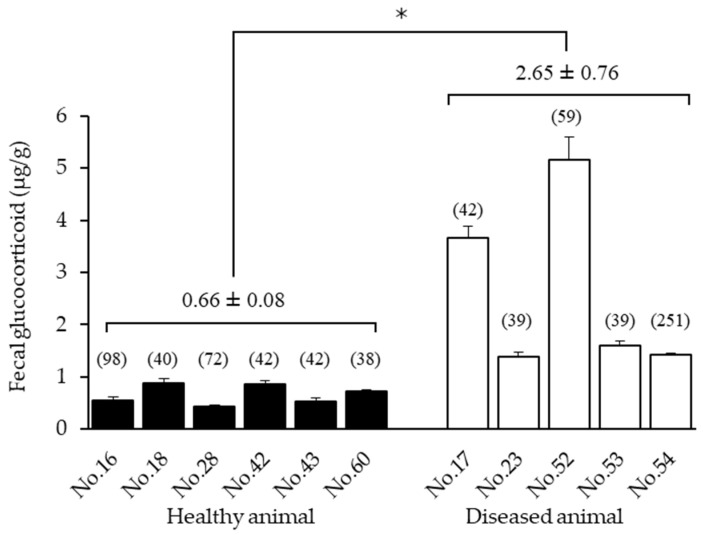
Average concentrations of fecal glucocorticoid metabolites for 6 healthy (No.16, No.18, No.28, No.42, No.43, and No.60) and 5 diseased (No.17, No.23, No.52, No.53, and No.54) Tsushima leopard cats. No17 and No.23: renal dysfunction; No.52: FIV and hernia; No.53: FIV and adrenal tumor; No.54: FeLV. Numbers in parentheses indicate the number of fecal samples analyzed. To avoid the effects of temporary stress, data from the day of anesthesia to the 4th day were excluded from each mean for the examined animals. * The diseased animals showed significantly higher values compared to the healthy animals (*p* < 0.05).

The mean concentrations of fecal GC metabolites in six seemingly healthy cats and five diseased cats were 0.66 ± 0.08 and 2.65 ± 0.76 μg/g, respectively. The diseased individuals showed significantly higher values in comparison to healthy individuals (*p* = 0.0185 < 0.05) (Figure 3). The mean concentration of fecal GCMs for No.52, which was diagnosed with disc herniation, showed 5.15 ± 0.05 μg/g, and the mean concentrations for No.17 and No.23, which suffered from renal dysfunction, showed 3.67 ± 0.21 and 1.38 ± 0.09 μg/g, respectively. Furthermore, No.53, which had the feline immunodeficiency virus (FIV), showed 1.61 ± 0.09 μg/g, and No.54, which had the feline leukemia virus (FeLV), showed 1.42 ± 0.04 μg/g.

### 3.3. Identification of Fecal Glucocorticoid Metabolites

**Figure 4:**
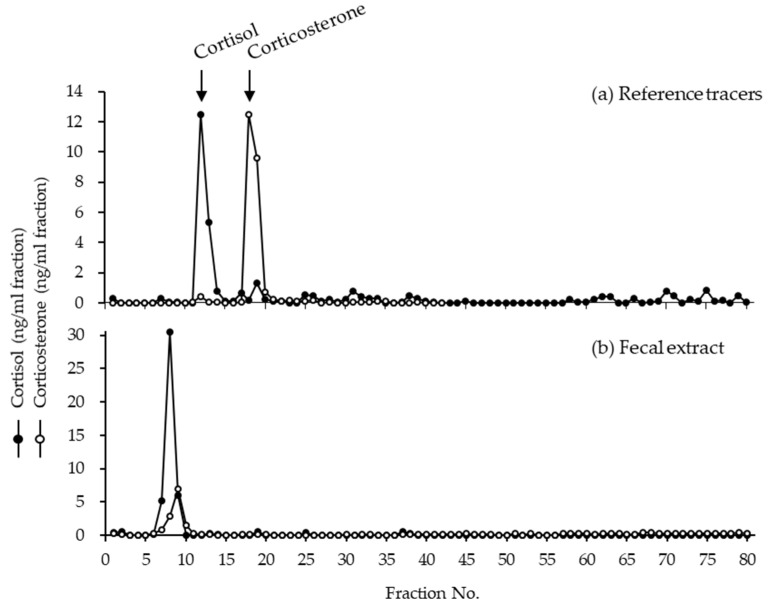
The immunoreactive cortisol and corticosterone in HPLC fractions of (**a**) reference tracers and (**b**) fecal extract from cat No.52. Top of the figure shows the immunoreactive peaks measured from the mixture of reference tracers by using cortisol and corticosterone antibodies. Bottom of the figure shows HPLC separation of immunoreactive glucocorticoid metabolites from feces. The arrows show the elution positions of the reference tracers for cortisol and corticosterone.

The immunoreactive cortisol and corticosterone in HPLC fractions of fecal extract from cat No.52 are shown in Figure 4. The measurement of cortisol in the fecal extracts shown a large peak at fraction No.7, and the measurement of corticosterone showed a peak at fraction No.9; however, the identity of the immunoreactive peaks could not be known. Clear immunoreactive peaks for the elution positions of cortisol (No.12–13) and corticosterone (No.18–19) were observed in the mixture of reference tracers, but not in the fecal extracts.

## 4. Discussion

In this study, changes in fecal GCM concentrations as useful indicators of adrenal activity or stress were investigated in the endangered Tsushima leopard cats for the first time.

After a physical examination under anesthesia, the fecal GC metabolite concentrations remarkably increased in four of the six cases. It is generally thought that restraint for blood collection is a stressor, and the concentration of circulating GC was noticeably increased within the first several minutes [26]. In the Tsushima leopard cats, restraint or blood collection also seemed to have influence on adrenal activities.

Physical pain appeared to cause stress because the individual diagnosed with disc herniation showed relatively high fecal GCM concentrations. Additionally, the stress derived from physical pain seemed to be relieved by the administration of steroids because the fecal GCM concentrations decreased after steroid administration.

It is generally thought that chronic glomerulonephritis, which is probably affected by the deposition of immunoconjugates somehow, is a major cause of developing renal dysfunction in dogs and cats [27]. Additionally, it is believed that the secretion of GC is enhanced by the secretion of cytokines, which are released by inflammation [10]. In the present study, the chronic increase in fecal GCM concentrations in Tsushima leopard cats suffering from renal dysfunction indicated that GC secretion was a biological response to the symptoms. Renal dysfunction of the domestic cat persisted over a period of several months to several years after initially showing symptoms, such as chronic glomerulonephritis; therefore, early prevention therapies are needed such as the restriction of phosphorus and the addition of fish oil in the feed [28]. The diagnosis of renal dysfunction in the early stages may be possible by monitoring the changes of fecal GCM concentrations in Tsushima leopard cats.

FIV is a virus that causes immunodeficiency, like how the human immunodeficiency virus (HIV) does, and FeLV is a virus that causes lymphosarcoma and leukemia. When domestic cats are infected with these viruses, the response can cause the inflammation of the gastrointestinal tract, ulcers, and other complications [29,30]. Additionally, it was reported that HIV infection changes the biological response of the corticotropin-releasing hormone (CRH) [31]. In this study, it seemed that the chronic increase in fecal GCM concentrations indicated the stress caused by the virus or by the inflammation derived from complications with the infection. There is a possibility that GC exacerbated the physical condition of the diseased individuals, because GC decreases the immune response to FeLV infection and decreases the antiviral activity of T-cells in response to FIV [29]. Thus, stress from physical pain combined with disease from virus infection has an enhanced negative influence on the living body.

The GCMs found commonly in fecal extracts were not identified, but they were determined to not be cortisol or corticosterone. In HPLC fractions obtained by Young et al. [20] from the feces of domestic cats, the cheetah, and the clouded leopard, GCMs were not identified; however, unknown metabolites common to these felid species were detected. In the present study, the Tsushima leopard cat was not an exception. Moreover, detection methods using the cortisol antibody were effective for monitoring adrenal activity, because increased fecal GCMs following ACTH administration were still observed in many felids [18,19]. Hence, GCM discharge into the feces of Tsushima leopard cats, like other felids, and the fecal assay using the cortisol antibody is useful for monitoring adrenal activities. 

## 5. Conclusions

The measurement of fecal GCMs by the use of a cortisol assay is useful for monitoring adrenal cortical function in the Tsushima leopard cat. The measurement of fecal GCM concentrations enabled insight into the stress states caused by restraint and physical examination under anesthesia, and this can facilitate the observation of therapeutic effects in individuals with renal dysfunction, infection, or pain. The routine monitoring of fecal GCM concentrations would improve the husbandry and physical health management of Tsushima leopard cats in captivity. 

## Figures and Tables

**Table 1:** Tsushima leopard cats measured fecal glucocorticoid (GC) in this study.

Studbook No.	Sex	Birth/Caught Date	Institute	Months of GC Measurement (Age Range)	No. of Samples Collected	Physical Exam Date
Healthy animals						
No.16	♂	CB: 7 April 2003	YZG	1.8 months (3.6–3.7) 1.0 month (7.0–7.1) 2.0 months (7.5–7.6)	38 28 40	- 1: 10 May 2010 2: 19 October 2010
No.18	♀	CB: 5 May 2003	FZG	6.7 months (3.0–3.6)	42	-
No.28	♂	CB: 19 April 2004	YZG	1.8 months (3.5–3.6) 1.2 months (6.0–6.1)	42 36	- 16 May 2010
No.42	♀	CB: 9 May 2007	TFPZ	4.6 months (2.6–2.9)	42	-
No.43	♂	WC: 12 May 2002	FZG	4.0 months (unknown)	42	-
No.60 (Mk−45)	♂	WC: 28 December 2009	FZG	4.8 months (unknown)	41	15 February 2010
Diseased animals						
No.17; renal dysfunction	♀	CB: 7 April 2003	TWCC	36.0 months (3.7–6.7)	42	-
No.23; renal dysfunction	♂	CB: 3 April 2004	TWCC	2.1 months (5.2–5.3)	42	29 June 2009
No.52 (Mt−09); FIV, hernia	♂	WC: 20 December 2000	TWCC	2.9 months (unknown)	59	31 January 2007
No.53 (CFT−17); FIV, adrenal tumor	♀	WC: 22 September 2002	TWCC	1.8 months (unknown)	42	31 October 2008
No.54 (Fm−28); FeLV	♀	WC: 14 March 2005	TWCC	1.8 months (unknown) 1.4 months (unknown) 2.0 months (unknown) 2.0 months (unknown) 1.7 months (unknown) 1.2 months (unknown)	36 42 61 56 42 30	1: 29 June 2007 2: 2 June 2008 3: 31 October 2008 4: 30 May 2009 5: 4 August 2010 6: 26 October 2010

FIV: feline immunodeficiency virus; FeLV: feline leukemia virus. CB: captive breeding; WC: wild caught (rescue). YZG: Yokohama Zoological Gardens ZOORASIA; TWCC: Tsushima Wildlife Conservation Center, Ministry of the Environment; FZG: Fukuoka City Zoological Garden; TFPZ: Toyama Municipal Family Park Zoo.

## Data Availability

Not applicable.
